# The Role of the Bacterial Flagellum in Adhesion and Virulence

**DOI:** 10.3390/biology2041242

**Published:** 2013-10-25

**Authors:** Johanna Haiko, Benita Westerlund-Wikström

**Affiliations:** Division of General Microbiology, Department of Biosciences, University of Helsinki, P. O. Box 56, FI-00014 University of Helsinki, Finland; E-Mail: johanna.haiko@helsinki.fi

**Keywords:** bacterial flagella, flagellin, FliD, adhesion, invasion

## Abstract

The bacterial flagellum is a complex apparatus assembled of more than 20 different proteins. The flagellar basal body traverses the cell wall, whereas the curved hook connects the basal body to the whip-like flagellar filament that protrudes several µm from the bacterial cell. The flagellum has traditionally been regarded only as a motility organelle, but more recently it has become evident that flagella have a number of other biological functions. The major subunit, flagellin or FliC, of the flagellum plays a well-documented role in innate immunity and as a dominant antigen of the adaptive immune response. Importantly, flagella have also been reported to function as adhesins. Whole flagella have been indicated as significant in bacterial adhesion to and invasion into host cells. In various pathogens, e.g., *Escherichia coli*, *Pseudomonas aeruginosa* and *Clostridium difficile*, flagellin and/or the distally located flagellar cap protein have been reported to function as adhesins. Recently, FliC of Shiga-toxigenic *E. coli* was shown to be involved in cellular invasion via lipid rafts. Here, we examine the latest or most important findings regarding flagellar adhesive and invasive properties, especially focusing on the flagellum as a potential virulence factor.

## 1. Introduction

Many Gram-positive and Gram-negative bacterial species and also Archaea as well as some eukaryotic cells have a flagellum (‘whip’ in Latin). Flagellum is primarily a motility organelle that enables movement and chemotaxis. Bacteria can have one flagellum or several, and they can be either polar (one or several flagella at one spot) or peritrichous (several flagella all over the bacterium). In addition to motility, flagella possess several other functions that differ between bacteria and during the bacterial life cycle: a flagellum can, for example, participate in biofilm formation, protein export, and adhesion. This review mainly focuses on the adhesive functions of flagella, and studies on the direct interaction of flagella with the host targets will be emphasized, though flagellar motility is often a prerequisite for adhesion and/or invasion. Duan *et al.* [1] have recently summarized in more detail the various roles of bacterial flagella in pathogenicity. For reviews on the role of flagella in biofilms, see Prüss *et al.* [2] and Conrad [3].

**Figure 1:**
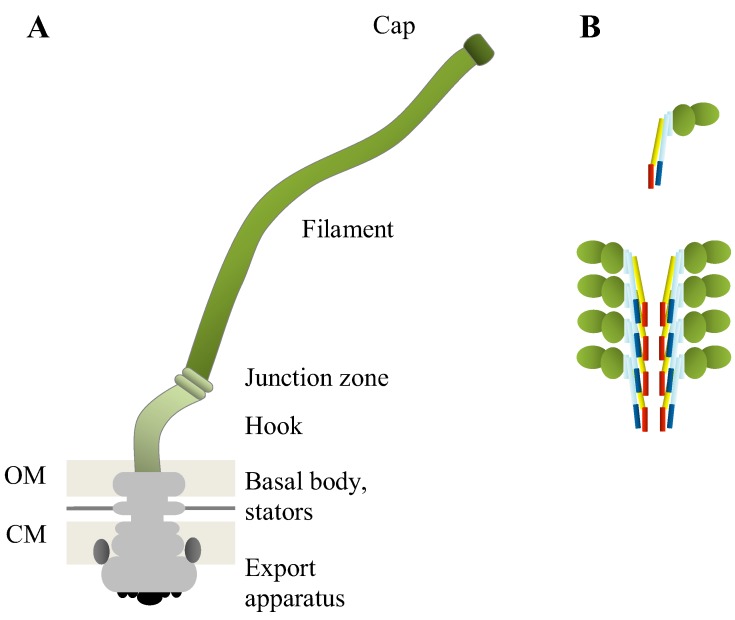
(**A**) Schematic presentation of bacterial flagellum structure. The pentameric FliD cap at the distal end of the filament, the hollow filament composed of about 20,000 identical flagellin subunits, the junction zone between filament and hook, and the hook connecting the filament to the basal body, represent extracellular parts (green shades) of the flagellum. The basal body (grey) in the cell wall consists of a centrally located hollow rod that connects different rings embedded in the outer membrane (OM), the peptidoglycan layer, and the cytoplasmic membrane (CM). Stator complexes (dark grey), composed of membrane proteins MotA and MotB, are associated with the CM-bound ring and the cytoplasmic ring below the CM, and provide motility-required energy. The cytoplasmic export machinery (black) that secretes the extracellular subunits is located within the cytoplasmic ring. Note that OM-associated parts of the basal body are absent in the flagella of Gram-positive bacteria. (**B**) Schematic presentation of flagellin monomer (upper panel) and flagellin polymerization (lower panel). The variable, exposed, globular domains of flagellin are shown in green. The conserved *N*- and *C*-terminal regions involved in flagellum polymerization are indicated (blue, light blue, yellow, red) as well as the regions binding to TLR5 (light blue, yellow) and those involved in inflammasome formation (blue, red).

The length of the typical flagellum of *Escherichia coli* is about 10 µm and the diameter is 20 nm. Over 60 structural and regulatory proteins are required for flagellum assembly and function. Flagellum consists of a cytoplasmic export apparatus, a basal body embedded in the cell membrane (CM), a hook that connects the basal body to the filament, and a filament that functions as a propeller (Figure 1A). Flagellar assembly starts with the CM-associated components of the basal body and the secretion apparatus, through which the other flagellar proteins are then secreted, first the remaining basal body components, then the hook and the hook-filament junction proteins. Filament assembly starts after the hook (120 FlgE molecules) has been completed and the filament capping proteins have been positioned. The filament is composed of about 20,000 flagellin (FliC) proteins that are incorporated below the distal pentameric FliD cap, which functions as a plug and is required for assembly of nascent monomeric flagellin. A two-component signaling cascade involving chemotaxis-related proteins affects flagellar rotation, which is facilitated by the engine consisting of the basal body-associated stator proteins and the basal body, which functions as a rotor. Some proteins relevant to this review and their functions are listed in Table 1. For a detailed description of flagellar assembly, see Chevance and Hughes [4]. For a review on chemotaxis, the reader is referred to Sourjik and Wingreen [5].

**Table 1:** Overview of flagella proteins relevant for the review ^(a)^.

Role in/Part of flagellum	Protein name/Number of proteins	Function
Regulation of flagellar biosynthesis	FlhC, FlhD, FlbA	Regulators
	FliI	Export-related ATPase
	FliA	Flagellar sigma factor
Motility/chemotaxis	CheA	Smooth swimming
	CheB	Tumbling
	CheW, CheV	Chemotaxis
	MotA, MotB	Rotation of flagellum
Export machinery	9 different proteins	Protein export
	FlhE	Chaperone or plug
Basal body	20 different proteins	Cell-wall anchor, rotor, holds export apparatus
	FlgJ	Muramidase
Hook	FlgE	Hook
Hook-filament junction	FlgK, FlgL	Connect filament to hook
Filament	FliC (alternative names FljB, FlaA, FlaB)	Main structural subunit
	FliD	Filament cap

^(a)^ Information combined from [2,4,5,6,7]. The designations used are mainly the names in enteric bacteria; for homologs in other species, see [8].

Bacterial adhesion is a critical initiation step in bacterial colonization and persistence, both for pathogens and commensals. Bacteria express various adhesive surface structures such as capsule, fimbriae or pili, and several surface proteins (for examples the reader is referred to Klemm *et al.* [9]). Typically the adhesive structures are not expressed at the same time as the flagellum, so that movement and attachment occur one at a time. Thus, bacteria switch from motile to sessile lifestyle and *vice versa*, and these changes are triggered by different environmental conditions—such as temperature, osmolarity, and pH—which regulate the expression of the *flhDC* flagellar master operon [2]. The regulation of flagellar expression occurs temporally at both the level of transcription and assembly [2,4]. The flagellum has also been shown to function as an export apparatus that mediates extracellular secretion of non-flagellar virulence-associated effector proteins and biotechnologically important heterologous polypeptides [1,10,11]. 

From the mammalian host perspective, the flagellum is relevant for immune defense: The immune system recognizes flagellin, which triggers adaptive and innate immune response. The conserved *N*- and *C*-termini of monomeric flagellin (Figure 1B) involved in flagella assembly interact with cytoplasmic NOD-like receptors in eukaryotic cells and induce the formation of inflammasome, which leads to pyroptosis [12,13,14]. Flagellin belongs to molecules containing a pathogen-associated molecular pattern (PAMP) that is recognized by toll-like receptor 5 (TLR5) [12]. TLR5 is mostly expressed at the basolateral surface of intestinal epithelial cells and by monocytes and fibroblasts, and binds the conserved termini of flagellin, which leads to the activation of cytokine secretion in host cells [12,15]. The central region of FliC is variable in sequence, and this region is exposed in native flagellin [16,17]. Sequence variation explains the observed differences in e.g., antigenic variation and the adhesive functions of flagellins from different bacterial strains or species (see text and Table 2 below).

**Table 2:** Direct and indirect roles of flagella in bacterial adhesion.

Role of flagella/Bacterial species	Flagellum/Protein/Gene	Effect/Role in virulence	Target/Administration route	Receptor	Reference
*Adhesion*					
*Bordetella bronchiseptica, Bordetella pertussis*	Flagellum	Adhesion	HeLa cells	ND	[18]
*Burkholderia* *pseudomallei*	Flagellum	Adhesion; invasion	*Acanthamoeba* *astronyxis*	ND	[19]
*Campylobacter* *jejuni*	Flagellum	Adhesion	Intestine-407	ND	[20]
*Clostridium* *difficile*	FliC, FliD; flagellum	Binding; no effect	Mouse cecal mucus, hamster	ND	[21,22]
*Escherichia* *coli*	Flagellum, FliC	Adhesion; Microcolony formation	HeLa cells	ND	[23]
	Flagellum, FliC	Binding	Mucins ^(a)^, bovine intestinal mucus, laminin, collagen	ND	[24]
	Flagellum	Adhesion; invasion	Polarized Caco-2BBe, T-84; BREC; Caco-2; T-84; IPEC-J2; IPEC-1	ND	[25,26,27,28,29,30]
	Flagellum	Association	HBMEC	ND	[31]
	Flagellum, FliC	Adhesion	Caco-2, mice intestine	EtpA	[32]
	FliC	Adhesion	Human intestinal cryosections, LS174T	gluconate	[33]
*Pseudomonas* *aeruginosa*	FliC	Adhesion; virulence	1HAEo; mice	GM1, GD_1a_, asialo-GM1	[34]
	FliC; FliD	Adhesion	mucin, MUC1	ND	[35,36,37]
	FliD	Adhesion	human respiratory mucin	Lewis x glycotype	[38]
	Flagellum	Adhesion	Calu-3 basolateral surface	HSPGs ^(b)^	[39]
*Salmonella* *enterica* serovar Dublin	Flagellum	Adhesion; virulence	Intestine-407; orally in mice	ND	[40]
*Salmonella* *enterica* serovar Typhimurium	Flagellum	Adhesion	Intestine-407	ND	[40]
*Stenotrophomonas* *maltophilia*	Flagellin	Adhesion	Mouse tracheal mucus	ND	[41]
*Motility*					
*Cronobacter* *sakazakii*	Flagellum	Adhesion; biofilm formation	Caco-2	ND	[42]
*Escherichia* *coli*	Flagellum	Mucus penetration; persistence; invasion	Hep-2, HT2916E, HT2919A; orally in chicks; HCT-8	asialo-GM1, lipid rafts	[43,44,45,46,47,48]
	FliC	Virulence	Orally in Sm-treated mice	ND	[49]
*Helicobacter* *pylori*	Flagellum	Colonization	Gastric epithelium, orally in mice	ND	[49]
*Proteus* *mirabilis*	Flagellum	Invasion; colonization	Human renal proximal tubular epithelial cells, EJ/28	ND	[50,51]
*Salmonella* *enterica* serovar Dublin	Flagellum	Increased invasion	Intestine-407	ND	[40]
*Salmonella* *enterica* serovar Enteritidis	Flagellum	Increased invasion	Caco-2, Hep-2, Div-1	ND	[52,53,54]
*Salmonella* *enterica* serovar Typhimurium	Flagellum	Increased adhesion; invasion	Intestine-407	ND	[40]
*Regulation*					
*Escherichia* *coli*	*flhDC*	Adhesion, invasion	Intestine-407	ND	[55]
	*flhC*	Colonization	Orally in cattle	ND	[56]
	FliA	Adhesion, invasion	Intestine-407	ND	[57]
*Helicobacter* *pylori*	*flbA*	Adhesion	Kato III, primary human gastric epithelial cells	ND	[58]

^(a)^ bovine submaxillary gland type I, porcine stomach type II; ^(b)^ heparan sulfate proteoglycans; ND, not determined.

The bacterial flagellum thus affects bacterial virulence in various ways, *i.e.*, by providing motility towards host targets, promoting early biofilm formation and thus bacterial survival, secreting virulence factors, triggering the adaptive and innate immune defense, and by promoting adherence and invasion.

## 2. Motility and Virulence

In many bacterial species, the flagellum is an acknowledged virulence factor, and non-flagellated strains have in several cases been observed to be less virulent. The flagellum can act directly as an adhesin, as detailed in Section 3 and Section 4, but can also affect virulence by other means. Motility towards a host cell is a prerequisite for adhesion and invasion, and flagella can play an essential role in colonization by facilitating bacterial motility even if the flagella do not directly participate in the adhesion or invasion. Flagella can also contribute to virulence by regulating the expression of other virulence factors [2] and, as discussed below, the flagellum in some cases affects virulence in more than one manner.

### 2.1. Flagellum Affects Virulence Mainly by Facilitating Motility

Early studies have shown that the single polar flagellum of *Vibrio cholerae*, the causative agent of cholera, is crucial for its virulence: non-motile *V. cholerae* had reduced virulence in mice, and their adsorption to cross sections of mice intestine was decreased compared to motile *V. cholerae* [59].

In *Salmonella enterica* serovar Enteritidis, a common cause of food-borne diarrhea, studies with non-flagellated Δ*fliC* and flagellated but non-motile *motA* mutants have shown that functional flagella enhance the invasive capacity of the bacterium into Intestine-407 and Caco-2 cells (see Table 3 for cell lines mentioned in the text) by enabling efficient motility, but the flagella filament *per se* is not required for adhesion and invasion [52,53]. However, non-flagellated mutants of *S.* Enteritidis have been shown to cause significantly less of the typical invasion-associated membrane ruffling than the wild-type strain in cultured human Hep-2 and avian Div-1 epithelial cells, indicating that flagella are involved in the early events of *S.* Enteritidis invasion in a still uncharacterized manner [54].

**Table 3:** Cell lines mentioned in the review ^(a)^.

Abbreviation	Cell line	Origin
1HAEo	airway epithelial cells	human
A549	lung epithelial cells	human
BREC	primary rectal epithelial cells	bovine
Caco-2	colorectal adenocarcinoma epithelial cells	human
Caco-2BBe	colorectal adenocarcinoma epithelial cells expressing enterocyte-like brush border	human
Calu-3	lung adenocarcinoma epithelial cells	human
CHO Lec-2	relatively ganglioside deficient CHO derivative	Chinese hamster
CHO-Muc1	mucin 1-expressing ovary cells	Chinese hamster
Div-1	gastrointestinal mucosal cells	avian
EJ/28	urothelial cells	human
16HBE	polarized airway epithelial cells	human
HBMEC	brain microvascular endothelial cells	human
HCT-8	ileocecal colorectal adenocarcinoma cells	human
HeLa	cervical tumor cells	human
Hep-2	epidermoid cancer cell line contaminated by HeLa cells	human
HT29	colorectal adenocarcinoma epithelial cells	human
HT2916E	mucus-secreting subclone of HT29	human
HT2919A	non-mucus-secreting subclone of HT29	human
Int(estine)-407	intestinal embryonic jejunum and ileum epithelial cells contaminated by HeLa cells	human
IPEC-1	neonatal ileal and jejunal epithelial cells	piglet
IPEC-J2	neonatal jejunal columnar epithelial cells	piglet
Kato III	gastric adenocarcinoma cells	human
LS174T	mucin-secreting colorectal adenocarcinoma epithelial cells	human
RAW264.7	macrophage cells	murine
T24	bladder epithelial cells	human
T-84	colorectal adenocarcinoma cells	human

^(a)^ Information collected from relevant original and review articles, and culture collections.

Recently, Olsen and colleagues [40] revealed serovar-specific differences in the involvement of flagella and chemotaxis genes in the adhesion and invasion of *Salmonella*. In the cattle-adapted *Salmonella enterica* serovar Dublin, the flagella filament was required for adhesion and invasion of Intestine-407 cells, whereas adhesion was unaffected in the constitutively tumbling *cheB* mutant and the constitutively smoothly swimming *cheA* mutant indicating that targeted motility is not essential for the adhesive capacity. In the broad-host-range *Salmonella enterica* serovar Typhimurium, adhesion to Intestine-407 was decreased by the deletion of flagellin and chemotaxis-related genes. Co-infection of mice with wild-type and mutant strains of both serovars revealed that the chemotaxis-related genes were dispensable *in vivo* in both serovars. The virulence of the *S*. Dublin *fliC* mutant was decreased with oral but not with intraperitoneal administration indicating that flagellin is important at the early colonizations stages of *S*. Dublin infection. In *S*. Typhimurium, deletion of the flagellin genes *fliC* and *fljB* rendered the mutant more virulent when administered intraperitoneally, whereas no effect was observed with oral infection indicating that flagellin is not required at the systemic phase of *S*. Typhimurium infection. Taken together, the role of flagella in *Salmonella* pathogenesis appears to be mainly related to motility, but flagellum affects adhesion and invasion in a serovar- and/or host-target dependent manner that reflects the lifestyles of the different serovars.

*Cronobacter* spp. are opportunistic food-borne pathogens that can cause serious diseases like bacteremia and meningitis in neonates. A study of a random transposon mutant library of the clinical *Cronobacter sakazakii* strain ES5 showed that peritrichous flagella are involved in biofilm formation on polystyrene and adhesion to Caco-2 cells [42]. In the study, five mutants carried transposons in flagellum-associated genes *fliD*, *flgJ*, and *flhE*. Phenotypically the *fliD* mutant had shorter flagella than the wild-type, the *flgJ* mutant lacked flagella, and the *flhE* mutant had flagella morphologically similar to the wild-type. Motility could not be examined, but the biofilm formation was drastically reduced in *fliD* and *flhE* mutants. Dramatic decrease in adhesion to Caco-2 cells was observed with *flgJ* mutant, and *flhE* mutant adhered as well as the wild-type, whereas the adherence of the *fliD* mutant was not measured. The results apparently indicate that motility is relevant in *C. sakazakii* adhesion to Caco-2 cells, but direct interaction of flagella and Caco-2 cannot be excluded.

*P.* *mirabilis*, a cause of urinary tract infections, requires peritrichous flagella for invasion into renal epithelial cells and urothelial cells, thus enabling colonization of the urinary tract of mice [50,51]. Contradictory findings of the importance of flagella in *P. mirabilis* pathogenesis have also been reported, indicating that a flagellum is not necessary in all infection models or for all *P. mirabilis* strains causing urinary tract infections [60,61], however nowadays flagella are regarded as a crucial virulence factor for *P. mirabilis* [62].

### 2.2. Flagellum Affects Virulence by Regulating Other Virulence Factors

Polar flagella are important virulence factors for *Helicobacter pylori*, a cause of gastric ulcers, because motility enables the bacteria to reach the gastric epithelium, adhere to it with several adhesins, and colonize the epithelium (reviewed by Sheu *et al.* [63]). Studies with a non-motile *fliD* mutant showed that FliD and thus a functional flagellum is required for the colonization of mice [64]. Clyne *et al.* [58] have studied whether *H. pylori* flagella are directly involved in adhesion by constructing flagellin (*flaA* and/or *flaB*) mutants and a flagellar regulator (*flbA*) mutant, and it appeared that all mutants adhered to gastric cells, indicating that flagella do not play a direct role in adhesion of *H. pylori* and suggesting that in addition to regulating flagella, FlbA may regulate some *H. pylori* adhesins.

## 3. Flagellum-mediated Adhesion

In several bacterial species, such as *E. coli*, *Pseudomonas aeruginosa*, and *Clostridium difficile*, the flagellum is multifunctional and plays a role in motility and is an adhesive organelle. A comprehensive review of studies on the direct role of the flagellum as an adhesin is presented below.

### 3.1. Escherichia coli

#### 3.1.1. *E. coli* Pathovars and Role of Flagella in Virulence

The Gram-negative enterobacterial species *E. coli* comprises a heterogeneous group of strains belonging on the one hand to commensals in the gut microbiota and on the other to pathogens. Two major groups of pathogenic *E. coli* exist, namely extraintestinal pathogenic *E. coli* (ExPEC) and intestinal pathogenic *E. coli* (IPEC). Human pathogenic *E. coli* strains are divided into pathovars on the basis of the symptoms and the disease they cause as well as some specific virulence determinants (Table 4). IPEC strains, which originate in domestic animals and infect human beings mainly via contaminated food or water, are divided into six main pathovars: enterohaemorrhagic *E. coli* (EHEC) causing outbreaks of severe gastroenteritis that can lead to haemorrhagic colitis or hemolytic-uremic syndrome; enteropathogenic *E. coli* (EPEC) which can cause diarrhea in young infants mainly in developing countries and also includes atypical enteropathogenic *E. coli* (aEPEC) that is an emerging enteropathogen; enterotoxigenic *E. coli* (ETEC) that causes diarrhea in infants and traveler’s diarrhea; enteroinvasive *E. coli* (EIEC) that causes dysentery; enteroaggregative *E. coli* (EAEC) that, depending on expressed virulence factors, can represent non-pathogenic strains or even cause hemolytic uremic syndrome; and diffusely adherent *E. coli* (DAEC), which causes diarrhea in young children [65]. The term Shiga-toxin producing *E. coli* (STEC) can include all variants of a particular pathovar, e.g., all EHEC strains produce the Shiga-toxin and are hence STECs, or only some types of a pathovar, e.g., Shiga-toxin producing EAEC strains are referred to as STEAEC. The Crohn’s disease associated pathovar is called adherent-invasive *E. coli* (AIEC), the avian pathogenic *E. coli* (APEC) resembles human ExPEC and causes colibacillosis in poultry, and fimbriated pathogenic *E. coli* is responsible for serious infections in piglets are pathovars not as well characterized as the IPECs and ExPECs [65,66,67,68,69].

*E. coli* express peritrichous flagella, which are classified into H-serotypes on the basis of sero-reactivity of the variable antigenic domain of FliC [70] (see Figure 1B). One pathovar may contain strains of different H serotypes and similar H serotypes can be found in more than one pathovar [69]. Early reports regarding the putative role of the flagella of APEC and Shiga-toxin producing EHEC in bacterial virulence indicated that *E. coli* flagella were not directly involved in bacterial adhesion but played a role in invasion. Experiments performed by inactivation of the flagellin gene and assessment of adhesive properties of the mutant in comparison to the wild-type strain indicated that the flagella of an APEC isolate did not significantly affect adhesion to Hep-2 cells and non-mucus-secreting HT2919A cells. The flagella mainly contributed to APEC penetration through the mucus in mucus-secreting HT2916E cells, and to persistence of APEC in the intestine of newly hatched chicks as well as of Shiga-toxin-negative EHEC O157:H7 in specific-pathogen-free chicks [43,44,45]. In Shiga-toxin producing EHEC O113:H21, a Δ*fliC* mutant colonized the intestine of streptomycin-treated mice as efficiently as the wild-type strain, but showed a reduced capacity to form the typical intimate association with colonic epithelium and was less virulent than the parental strain [49]. The flagellin of STEC O113:H21 was reported as essential for bacterial invasion into HCT-8 cells in a TLR5-, MyD88-, and IRAK-independent manner, although STEC adhesion was not mediated by FliC [47]. The results indicated that STEC interacted with lipid rafts, as pretreatment of HCT-8 monolayers with anti-asialoganglioside antibodies, cholesterol-depleting methyl-β-cyclodextrin, or tyrosine kinase-inhibiting genistein prior to invasion reduced STEC invasion. Invading STEC were also localized in the regions of lipid raft markers on HCT-8 cells. However, the pretreatment of HCT-8 cells reduced invasion of the STEC Δ*fliC* mutant, and the authors concluded that bacterial interaction with lipid rafts was not dependent solely on flagellin [47]. Conclusively, these aforementioned findings regarding APEC and especially Shiga-toxin producing EHEC O113:H21 indicate that flagellum-mediated motility is crucial in invasion, but that flagellin is not required for adhesion.

**Table 4:** *E. coli* pathovars relevant for the review.

Pathovar	Abbreviation	Disease/symptoms	Major site of colonization	Reference
*IPEC*				
Enterohaemorrhagic *E. coli*	EHEC	Gastroenteritis; hemorrhagic colitis; HUS ^(a)^	Large bowel	[65,69]
Enteropathogenic *E. coli*	EPEC	Infant diarrhea in developing countries	Small bowel	[65,69]
Atypical enteropathogenic *E. coli*	aEPEC	Persistent diarrhea in children	Small bowel	[69]
Enterotoxigenic *E. coli*	ETEC	Infant diarrhea; travelers’ diarrhea	Small bowel	[65,69]
Enteroinvasive *E. coli*	EIEC	Dysentery	Large bowel	[65,69]
Enteroaggregative *E. coli*	EAEC	Variable (from non-symptomatic to HUS)	Large bowel, small bowel	[65,69]
Diffusely adherent *E. coli*	DAEC	Diarrhea in young children; nosocomial adult diarrhea	Small bowel	[65,69]
Shiga-toxin producing *E. coli*	STEC	Dependent on Shiga-toxin expressing pathovar	Dependent on pathovar	[69]
*ExPEC*				
Newborn-meningitis causing *E. coli*	NMEC	Newborn meningitis; septicaemia	Brain, blood	[65,68,71]
Uropathogenic *E. coli*	UPEC	Urinary tract infections; septicaemia	Bladder, kidney, blood	[65,68]
*Other*				
Adherent-invasive *E. coli*	AIEC	Crohn’s disease	Ileum of small bowel	[65,67,68,69]
Avian pathogenic *E. coli*	APEC	Avian colibacillosis	Systemic infection	[67,68]
Porcine pathogenic *E. coli*	Porcine STEC	Post-weaning diarrhea; edema disease	Intestine;vasculature	[28,29,48]

^(a)^ hemolytic-uremic syndrome.

#### 3.1.2. Flagellum-mediated IPEC Adhesion

Several reports, however, show that flagella of some *E. coli* pathovars do mediate direct bacterial adhesion, and the majority of these reports focus on IPEC strains. Girón and colleagues [23] have shown that wild-type EPEC expressing H2 or H6 flagella adhere to HeLa cells and form microcolonies on them. Insertional inactivation of the *fliC*_H6_ gene in EPEC significantly decrease the adhesion, whereas the flagellated but non-motile isogenic *motB* mutant adheres to HeLa as efficiently as the wild-type strain thus demonstrating that the flagellar filament indeed is involved in adhesion, not only in the movement towards a target. Complementation of the *fliC* mutation with plasmid-cloned *fliC*_H6_ partially restored the adhesiveness of the mutant, and expression of FliC_H6_ in a non-adhesive laboratory strain of *E. coli* rendered it adhesive to HeLa cells. The interaction with HeLa cells was also demonstrated with purified H6 flagella, whereas purified H7 flagella did not bind to the cells. It was also shown that flagella expression in all motile EPEC strains tested (expressing e.g., H2, H6 and H34 flagella) was enhanced when the bacteria were grown on eukaryotic cells and that the flagella extended within and between the bacterial microcolonies. In a non-motile EPEC strain, low-level flagella expression was observed as well, whereas EHEC carrying the genes encoding H7 flagella and ETEC having the *fliC*_H9_ gene did not express flagella when the bacteria were grown on HeLa cells. Flagella of aEPEC O51:H40 were also observed to be adhesive: the wild-type aEPEC strain adhered to the colorectal cell line T-84, and invaded T-84 and Caco-2 cells, whereas an isogenic *fliC* mutant showed a significantly reduced adhesive and invasive capacity [27]. Taken together, the reports show that EPEC and aEPEC, but not EHEC (O157:H7) and ETEC (O8:H9), adhere to HeLa, T-84 and Caco-2 cells and that EPEC express flagella efficiently when cultured on HeLa cells. The authors speculate that a soluble molecule of eukaryotic origin regulates flagella production and thereby flagellum-mediated adhesion in EPEC.

Using immunodot analysis, Erdem and colleagues [24] showed that purified flagella and denatured monomeric flagellin of both EPEC O127:H6 and EHEC O157:H7 bound to immobilized commercially available bovine and porcine mucins, as well as to mucus prepared from bovine colon in a dose-dependent manner. Preincubation of purified flagella with commercial mucin followed by molecular exclusion chromatography demonstrated simultaneous elution of flagella and mucin, thus verifying the binding of flagella to mucin. The adhesive capacity of FliC-deficient mutants of EPEC O127:H6 and EHEC O157:H7 to bovine intestinal tissue was significantly lower than that of corresponding isogenic wild-type strains, thus indicating that FliC_H6_ but also FliC_H7_ mediate bacterial adhesion to bovine submaxillary gland mucin and porcine stomach mucin. Differences between H6 and H7 flagella in the interactions with host molecules were also observed: Purified H6 flagella bound to extracellular matrix proteins collagen and laminin, whereas H7 flagella did not interact with these proteins. Also, EHEC O157:H7 cells agglutinated rabbit erythrocytes independently of mannoside-binding type 1 fimbriae typically expressed by *E. coli*. The hemagglutination (HA) was inhibited by anti-H7 antibodies and mucins (see above), whereas a number of characterized carbohydrates and glycoproteins had no inhibitory effect. Moreover, the FliC-deficient isogenic mutant lost the HA capacity, and similar HA properties were not observed with EPEC O127:H6 or purified H6 flagella. The authors speculate that the observed mucin-binding property of H6 and H7 flagella may favor intestinal colonization, whereas the adhesion to the extracellular matrix proteins by H6 flagellum may contribute to colonization at sites where the intestinal barrier is disrupted. The relevance of the flagella-mediated HA property of EHEC remains to be elucidated, but apparently it reflects the receptor specificity of H7 flagella.

The adhesive properties of H7 of EHEC to BREC, which is the colonization site in cattle, were studied by Mahajan and colleagues [26]. They demonstrated that wild-type EHEC O157:H7 adhered to BREC, that adhesion of an isogenic *fliC* deletion mutant was significantly decreased, and that adhesion of the *fliC* mutant was restored by *trans* complementation with *fliC*_H7_ but not with *fliC*_H6_. The wild-type adhesion was blocked by anti-FliC_H7_ antiserum and by purified H7 flagella. Purified H7 flagella of EHEC bound directly to BREC, whereas EHEC flagella of serotypes H11 and H21 did not interact with bovine cells as assessed by indirect immunofluorescence microscopy. EHEC O157:H7 expressed flagella when applied onto BRECs, but indirect immunofluorescence microscopy and in-cell Western assay revealed a temporal expression of H7 flagella; the flagella were present during early colonization and on bacteria not associated with epithelial cells, but expression of flagella was repressed on bacteria in microcolonies or bacteria associated with the typical attaching and effacing lesions formed by EHEC on BRECs. The results are in good agreement with the study by Girón and coworkers [23] showing that EPEC, but not EHEC, express flagella when grown on cultured epithelial cells, and with the reports by Luck *et al.* [46] and Rogers *et al.* [47] that state that H21 flagella do not mediate adhesion to cultured epithelial cells. The results indicate that H7 flagella act as adhesins at the initiating phase of the infection, but are not required at later stages of EHEC infection. The preceding reports further demonstrate the relevance of the appropriate selection of host cells prior to the analysis of flagellar adhesion.

#### 3.1.3. Flagellum-mediated ExPEC Adhesion

The major ExPEC pathovars are uropathogenic *E. coli* (UPEC) causing urinary tract infections and the newborn-meningitis causing *E. coli* (NMEC), and more recently, APEC that have been shown to closely resemble ExPEC [67,68]. In contrast to IPECs, NMEC strains are associated with high mortality rates, are genetically closely related, express a number of well-characterized fitness factors and belong to only a few serotypes of which O18:K1:H7 is widely spread and the most studied serotype [71]. Transcriptome analysis has revealed that the expression level of the *fliI* gene is significantly increased in NMEC associated with HBMEC cells compared to NMEC not associated with cultured cells [72]. Further analysis indicated that the H7 flagellum of *E. coli* O18:K1:H7 may be involved in NMEC pathogenesis. Flagella-deficient (Δ*fliI*, Δ*flhDC*, and Δ*fliC*) mutants associated to HBMECs and invaded them significantly less efficiently than the isogenic wild-type strain. Further, the mutants complemented with plasmid-cloned *fliI*, *flhDC* and *fliC*, and the mutants (Δ*cheW*, Δ*motAB*) carrying non-motile flagella, associated with and invaded HBMECs equally well as the wild-type strain [31]. Also, the association rate of the wild-type NMEC strain was reduced to 40% after the treatment of HBMEC with purified recombinant flagellin, indicating that purified flagella block flagellum-mediated interaction of NMEC with HBMEC. Deletion of *fliC* did not affect the expression of Type 1 fimbriae, previously shown to interact with HBMEC [73]. Taken together, the results thus indicate that motility *per se* is not a key determinant in the association with and invasion into HBMECs by NMEC, but presence of the flagellar filament is apparently required for the interaction of NMEC with HBMEC, and FliC may, in concert with other virulence factors, play a role in meningitis caused by *E. coli* O18:K1:H7. Interestingly, according to the reports above, expression of H7 flagella of both EHEC and NMEC is upregulated upon contact with putative host cells.

#### 3.1.4. Role of Flagellum in AIEC Adhesion

In the AIEC type strain LF82, the flagellar master operon *flhDC* reduces bacterial adhesion to Intestine-407 cells indirectly by down-regulating the expression of adhesive Type 1 fimbriae, but flagella also affect bacterial invasion in a manner not restricted to motility or the presence of a flagellar filament [55]. Similarly, the *flhC* gene, but not the flagellin gene, reduces in an unknown manner the colonization of cattle by Shiga-toxin-producing EHEC O157:H7 [56]. Claret and colleagues [57] have shown that the flagellar sigma factor FliA regulates the Intestine-407 adhesion and invasion by strain LF82 via a cyclic-di-GMP-dependent pathway that down-regulates expression of Type 1 fimbriae. In addition to the indirect effect, the flagellum can also directly be involved in AIEC adhesion and invasion. Two characterized AIEC strains, LF82 and O83:H1, isolated from Crohn’s disease patients, weakly adhered to Caco-2BBe cells and invaded both Caco-2BBe and T-84 cells. On the other hand, two uncharacterized AIEC isolates, a laboratory *E. coli* HS strain used as a negative control, and an isogenic *fliC* mutant of AIEC O83:H1, were non-adhesive and non-invasive, whereas complementation of the *fliC* mutation fully restored the adhesiveness to intestinal epithelial cells and increased invasion of the complemented strain in comparison to the *fliC* mutant [25]. Conclusively, AIEC flagella contribute to bacterial adhesion and invasion indirectly by regulating expression of other adhesins but also directly by facilitating FliC-mediated adhesion in a strain-dependent manner.

#### 3.1.5. Flagellum-mediated Adhesion of Porcine Pathogenic *E. coli*

Porcine pathogenic *E. coli* frequently express various types of fimbriae, such as F18 and F4 fimbriae [66], which mediate bacterial adhesion to host cells. Recently, the role of flagellum as a virulence factor has been studied in more detail in porcine isolates. Duan and colleagues [28,29,48] have reported that *fliC* deletion mutants of the swine edema-disease causing STEC (O139:H1:F18ab) and post-weaning diarrhea (PWD) causing *E. coli* (O157:H019:F18ac) show reduced adhesion and invasion into IPEC-J2 and IPEC-1 cells compared to the isogenic wild-type strains. The adhesive phenotype of the mutants was restored by complementation of the deletion with plasmid-cloned *fliC*, and specific inhibition of bacterial adhesion by purified flagella further demonstrated the adhesive role of flagella. As in AIEC, regulatory cross talk between adhesive organelles was also indicated in porcine *E. coli* isolates. Type 1 fimbria expression was upregulated in the F18 Δ*fliC* mutants and in ETEC O8:F4ac, another cause of PWD, the F4-fimbria appeared to be the main adhesin whereas flagella mainly affected expression of the fimbriae [30,48]. The results thus show that flagella of porcine-pathogenic *E. coli* possess similar adhesive, invasive and regulatory properties to flagella of IPEC and ExPEC.

#### 3.1.6. Flagellum-mediated Adhesion of Probiotic *E. coli*

Bacterial adhesion to host surfaces is not always related to infection. In the human intestine, the microbiota and to some extent also probiotic bacterial strains protect the host surfaces against harmful intruders [74]. In order to successfully colonize the gut, probiotic bacteria should be able to adhere to intestinal surfaces. *E. coli* Nissle 1917 is a probiotic strain well-characterized regarding fimbrial genes and known to express H1 flagella, but other adhesive properties are not known [33]. A hyperflagellated variant of Nissle 1917 carrying up to 12 flagella per cell was assessed along with the wild-type strain, a Δ*fliC* mutant, and fimbriae-deficient mutants for adhesion to human intestinal cryosections. The hyperflagellated strain adhered efficiently, whereas the Δ*fliC* mutant showed no adhesion, and deletion of fimbriae genes had no impact on the adhesion capacity of Nissle 1917. More detailed analysis of the adhesive properties of Nissle 1917 indicated that the strain attached in a FliC-dependent manner to mucin-producing LS174T cells, but not to Caco-2 and T24 cells nor to immobilized murine mucus. Adhesion of wild-type Nissle 1917 and the hyperflagellated variant to intestinal cryosections was efficiently inhibited by mucin II, and flagella isolated from the wild-type strain bound to mucin II as well as human mucus. For identification of the receptor molecule of Nissle 1917 flagellum, isolated flagella were preincubated with carbohydrates known to be constituents of mucin II, and assessed for binding to immobilized mucin II and human mucus. In both cases, the binding was reduced only in the presence of gluconate. The adhesion of wild-type and hyperflagellated Nissle 1917 to human intestinal cryosections and immobilized mucin II was also inhibited by 100 mM gluconate, indicating that gluconate functions as a receptor for Nissle 1917 flagella in mucus. The results show that not only pathogenic but also probiotic *E. coli* possess adhesive flagella, and further studies are required to reveal the prevalence and biological significance of flagellum-mediated direct adhesion to host cell surfaces.

#### 3.1.7. Flagellum-mediated Indirect *E. coli* Adhesion

Flagella can also mediate bacterial adhesion indirectly via other molecules. EtpA is a secreted ETEC adhesin shown to bind to Caco-2 cells and mucin-expressing regions of mouse small intestine [32]. Adherence of ETEC to Caco-2 cells requires EtpA as well as flagella, since deletion of *etpA* decreased and deletion of *fliC* abolished the adhesion. Complementation of the *fliC* deletion with plasmid-cloned *fliC* restored the adhesive capacity to the level of the isogenic wild-type ETEC strain. Purification of EtpA revealed co-isolation of flagellin, regardless of flagellar serotype, indicating an interaction between the two types of molecules. Pull-down experiments showed that the toxin recognized the conserved *N*-terminus of monomeric FliC and bound to it and *in situ* immune electron microscopy revealed localization of EtpA to broken flagellar tips lacking FliD. Conclusively, EtpA and intact flagella together form a prerequisite for efficient adhesion of ETEC to intestinal cells and mouse small intestine.

As evident from the examples above, flagella of *E. coli* can be involved directly in bacterial adhesion and/or invasion or indirectly via another bacterial protein, such as EtpA in ETEC, or due to flagellar regulation of other bacterial adhesive/invasive factors. Frequently, the flagellum-associated adhesive/invasive property is closely related to specific *E. coli* pathovars.

### 3.2. Pseudomonas aeruginosa

*P. aeruginosa* is an opportunistic pathogen that can cause urinary tract infections and conditions such as pneumonia, particularly in cystic fibrosis patients. *P. aeruginosa* has a single polar flagellum that is crucial for motility and chemotaxis. The role of *P. aeruginosa* flagellum in adhesion has been investigated in several publications, and mucin is thought to be important target for *P. aeruginosa* adhesion as the lung epithelium contains a thick mucus layer.

*P.* *aeruginosa* Δ*fliF*, a mutant without flagellar membrane and supramembrane ring, has been reported to be defective in the adhesion to mucin [35]. However, as FliF is embedded in the outer membrane, it cannot act as an adhesin, and thus the adhesion-enabling function must be indirect rather than FliF-mediated. Later Arora *et al.* [36] reported that purified flagellar cap protein FliD inhibited the adhesion of non-piliated *P. aeruginosa* PAK to mucin from human sputum. They also showed that Δ*fliD* mutant did not bind mucin, and complementation with *fliD* restored the mucin binding ability, suggesting that FliD might mediate adhesion to mucin. Wild-type *P. aeruginosa* PAO1 has been shown to bind human airway mucins and Lewis x glycotypes on human respiratory mucins, while the Δ*fliC* and Δ*fliD* mutants do not [38]. Also in this study, complementation with *fliD* restored adherence while complementation with *fliC* was not performed. However, as flagellar biosynthesis requires FliD for the filament polymerization, it remains unclear whether FliD alone is responsible for adhesion or whether FliC or some other flagellar component has a role in adhesion as well. Direct interaction between FliD and mucins was not shown [36,38]. FliD of *P. aeruginosa* PAK mediated adhesion to mucin from human sputum [36], but seemed not to be involved in airway mucin binding [38], which may be due to the expression of distinct FliD types by the PAK and PAO1 strains [75].

Lillehoj *et al.* [37] showed that *P. aeruginosa* PAK adhered to MUC1 mucin expressed by CHO cells (CHO-Muc1), and that deletion of *fliC* abolished the adhesion. Purified flagellin and flagellin antiserum inhibited adhesion of *P. aeruginosa* to CHO-Muc1 cells. Surprisingly however, deletion of *fliD* did not affect binding, although flagellin filament cannot polymerize without FliD. It is therefore possible that *P. aeruginosa* also possess other mucin adhesins than flagellum. *P. aeruginosa* PAK Δ*fliC*, a derivative of a clinical isolate, has also been shown to be impaired in adhesion to 1HAEo cells compared to wild-type strain [34] and the Δ*fliC* mutant showed reduced virulence in a mouse model of pneumonia. Purified flagellin of *P. aeruginosa* bound to monosialylated glycolipid GM1 that is common in cell membranes, disialylated ganglioside GD_1a_—the major ganglioside in mammalian brain- and to asialo-GM1 present in several immune cells, for example [34].

In a recent study, *P. aeruginosa* PAO1 flagellum was shown to bind to heparan sulfate chains of heparan sulfate proteoglycans at the basolateral surface of polarized Calu-3 cells [39]. This was demonstrated with bacteria and flagella-coated fluorescent beads. The same study also showed evidence for the adherence of Type IV pili (Tfp) to *N*-glycans at the apical surface of the polarized lung epithelial cells. Subsequently, the adhesion of *P. aeruginosa* by flagellum or Tfp was shown to lead to changes in host signal transduction pathways by inducing phosphatidylinositol 3-kinase (PI3K)/Akt pathway. The activation of PI3K/Akt pathway led to bacterial entry into epithelial cells, and inhibition of PI3K decreased invasion but not adhesion at both apical and basolateral surfaces. Phosphorylation of Akt, a PI3K effector, was increased by flagellum- or Tfp-mediated binding. The flagellum-mediated adherence at the basolateral surface occurred via the epidermal growth factor receptor that is phosphorylated by flagellum, but Tfp-mediated adherence is independent of it. The ability to adhere and invade at both the basolateral and apical surfaces of epithelial cells may be crucial in the pathogenesis of *P. aeruginosa*. Flagellar component(s) responsible for the interaction with heparin sulfate remain(s) to be determined.

### 3.3. Clostridium difficile

The anaerobic spore-forming Gram-positive bacterium *C. difficile* is an opportunistic pathogen causing antibiotic-associated diarrhea especially in health-care environments. *C. difficile* carry peritrichous flagella, but the role of the flagellum in virulence has not been widely studied and reports on the adhesive properties of *C. difficile* flagella are partially contradictory. Thus, current results regarding the role of *C. difficile* flagella in virulence require further investigation. The early report by Tasteyre and colleagues [21] showed that *C. difficile* crude flagella extracts as well as purified recombinant FliD and FliC proteins bound in immunodot analyses to immobilized axenic mouse cecal mucus, but not to porcine stomach mucin. On the other hand, Dingle and colleagues [22] demonstrated, using wild-type *C. difficile* as well as isogenic *fliC* and *fliD* mutants, that the mutants adhered more efficiently to differentiated Caco-2 cells than the wild-type strain. In a hamster model FliC and FliD were nonessential for cecal colonization, but as bacterial growth curves indicated that the mutants grew more slowly and produced larger amounts of toxin than the wild-type strains, final conclusions regarding the role of *C. difficile* flagella in colonization cannot be drawn. Recent transcriptional analysis of the genome-sequenced *C. difficile* has demonstrated a differential expression of flagella genes during heat shock conditions; *fliC* was down-regulated, the level of *fliD* remained unchanged, and genes encoding the hook, rod, and basal body were up-regulated [76]. The authors speculated that motility may be down-regulated, but adhesion enhanced during infection. It is obvious that much of the data in the older papers is misleading.

### 3.4. Flagellar Adhesion of Additional Bacterial Species

In addition to the bacterial species already mentioned, the role of flagella in adhesion has been studied for example in *Burkholderia pseudomallei* that causes melioidosis, *Burkholderia cepacia*, an opportunistic pathogen, *Bordetella bronchiseptica*, which can colonize the respiratory tract and cause canine and porcine bronchitis, *Bordetella pertussis*, which causes whooping cough, the gastroenteritis-causing *Vibrio vulnificus* and *Campylobacter jejuni*, and the opportunistic pathogen *Stenotrophomonas maltophilia*.

*B.* *pseudomallei* is a facultative intracellular bacterium that is able to invade several cell types and survive inside macrophages and free-living amoebae [77,78]. The flagellum has been shown to mediate adhesion of *B. pseudomallei* to *Acanthamoeba astronyxis*, and the *fliC*-lacking mutant did not adhere or become internalized, even if the bacteria were centrifuged onto the amoebae [19]. Other studies have also been performed to investigate the adhesive properties of *B. pseudomallei* flagella, but in most cases, the flagellum does not seem to be the adhesin, but instead promotes motility toward targets. For example, flagella facilitated the invasion of *B. pseudomallei* into the mouse macrophage cell line RAW264.7 and human lung epithelial cell line A549, but flagella were not the only component responsible for bacterial invasion into epithelial cells and were not shown to contribute to adhesion [79]. The same study also compared the FliC of *B. pseudomallei* to FliC of a closely related but avirulent *B. thailandensis*, which has a 15 bp deletion in the variable region of *fliC* [80], but complementation of *B. pseudomallei* Δ*fliC* with *B. thailandensis fliC* improved invasion as much as complementation with *fliC* from *B. pseudomallei* [79]. No differences were observed between the Δ*fliC* mutant and the other strains, including *B. thailandensis*, when the bacteria were centrifuged to bring them in contact with the eukaryotic cells. Thus it must be noted that invasion ability seems to be correlated with motility, but other proteins than flagella probably mediate adhesion and invasion by *B. pseudomallei*, except in the case of amoeba adhesion. *B. cepacia*, which can cause disease in immunocompromised persons, particularly respiratory tract infections in cystic fibrosis patients, has polar flagella composed of flagellin that exhibits high sequence homology to *B. pseudomallei fliC* [81]. The invasiveness of *B. cepacia* into respiratory cells was dependent on functional flagella and motility, but flagella did not function as adhesins [82] thus resembling the pattern observed with *B. pseudomallei*. 

*Bordetella* species are pathogens, which colonize the respiratory tract, and their virulence factors, including flagella, are regulated by the *bvg* locus. Flagella of *B. bronchiseptica* and *B. pertussis* may contribute to bacterial adhesion to HeLa cells, shown also with purified *B. bronchiseptica* flagella [18].

*S.* *maltophilia* is frequently associated with respiratory tract infections, and it has been found to adhere to mouse tracheal mucus via flagella, shown with flagella-expressing clinical isolates and flagellin isolated from *S. maltophilia* [41].

*V.* *vulnificus* has a polar flagellum, and a non-motile flagellum-deficient hook mutant Δ*flgE* was less adherent to Intestine-407 cells, formed less biofilm, and the lethal dose in mice was increased [83]. The results thus indicate that the flagellum plays a role in *V. vulnificus* virulence.

*C.* *jejuni* adhered to Intestine-407 cells with flagellum and LPS, and purified *C. jejuni* flagella bound to epithelial cells but not to intestinal mucus [20]. *C. jejuni* has two highly (95%) identical flagellin genes, *flaA* and *flaB*, but only *flaA* is required for flagella-mediated motility and adhesion [84]. Motility was suggested to be important for invasion into Intestine-407 cells, but direct adhesive function of flagellum was not observed [84]. According to Grant *et al.* [85], flagella of *C. jejuni* are not required for adhesion to epithelial cells but are involved in invasion. This might be explained by FlaC, a protein secreted via flagella and found to increase invasion of *C. jejuni* to Hep-2 cells [86], or by other secreted proteins. FlaC is homologous to flagellin proteins FlaA and FlaB at *N*- and *C*-terminal regions but lacks the central domain and is not required for the expression of the functional flagellum [86].

## 4. Additional Adhesion-related Mechanisms of Flagella

The flagellum has an important role in biofilm formation, and biofilms on abiotic surfaces pose a remarkable health threat in cases such as clinical catheters and cooling systems. To explore in detail the adhesion and thereby formation of biofilms on catheters, the mechanism of flagellum binding was studied by Friedlander and colleagues [87]. UPEC were cultured on flat silicon surfaces as well as on patterned surfaces covered with bumps and submicrometer crevices too narrow to fit the bacterial cells, and the surfaces were analyzed for bacterial attachment and presence of flagella by scanning electron microscopy. During the first two hours after inoculation, adhesion to flat surfaces was more efficient, but after a longer incubation period, the colonization of the patterned surface was more efficient. Adherent bacterial cells were surrounded by flagella, as demonstrated by site-specific mutagenesis and scanning electron microscopy. The non-flagellated Δ*flhDC* and Δ*fliC* mutants showed reduced adhesion in comparison to the wild-type strain, whereas the flagellated paralyzed Δ*motB* mutant colonized the patterned surface as efficiently as the wild-type strain. The results indicate that flagella reach crevices, grasp to improve bacterial adhesion, and are able to penetrate substructures not accessible to the bacterial cells, as was described also for EPEC adhesion to HeLa cells and EHEC interaction with BRECs [23,26].

As *Salmonella enterica* serovar Typhi is frequently associated with cholesterol-rich gallstones in chronic carriers, the adhesion of *Salmonella* and formation of biofilm on cholesterol have been studied in more detail [88]. When a library of random transposon mutants of *S*. Typhimurium was assessed for adherence to cholesterol, mutants carrying transposons in flagellum-related genes showed reduced adhesion to cholesterol in comparison to the wild-type strain. The importance of the flagella filament in cholesterol-binding and biofilm formation on cholesterol was demonstrated by the inability of the non-flagellated *ΔflhC* mutant to form a biofilm, whereas the flagellated but non-motile *motA* deletion mutant formed biofilm on cholesterol as efficiently as the wild-type strain. Further, formalin-killed wild-type flagellated *S*. Typhimurium adhered to immobilized cholesterol as efficiently as live bacteria and functioned as a scaffold for biofilm formation of living flagellated as well as non-flagellated *Salmonella*. Adhesion to cholesterol by a Δ*fliC* mutant (expressing the FljB flagellin) was significantly reduced compared to the adhesion of Δ*fljB* mutant (expressing FliC flagellin) or the wild-type strain. Conclusively, flagella composed of FliC-type flagellin mediate adhesion to cholesterol in *S*. Typhimurium and promotes the early formation of biofilm. 

Flagella composed of polymeric flagellin do not bind to TLR5, whereas monomeric flagellin induces a TLR5-mediated inflammatory response [12]. Interestingly, Subramanian and Quadri [89] reported that host cell-produced lysophospholipids induced the secretion of biologically active, monomeric flagellin in *S.* Typhi and *S*. Typhimurium*.* The results were further supported by the observations that externally added lysophospholipid triggered secretion of monomeric flagellin in salmonellae and flagellin secretion was reduced in *Salmonella* if host cells were pretreated with inhibitors of lysophospholipid synthesis. The secretion of monomeric flagellin was not due to depolymerization of flagella filaments but a result of eukaryotic cell-induced flagellin expression in *S*. Typhi, and the results demonstrated that secretion of monomeric flagellin was dependent on cAMP-dependent signaling. The authors speculate that during intestinal infection, *Salmonella* can sense lysophospholipids produced by host epithelial cells, activate the export of monomeric flagellin, and thereby modulate the TLR5-mediated innate immune response, which could promote bacterial dissemination.

Gangliosides have been reported to act as receptors for flagellin, but also appear to facilitate flagellin-mediated signaling in eukaryotic cells. Purified *P. aeruginosa* PAO flagellin has been shown to bind glycolipid receptors (including GM1 and asialo-GM1) on CHO Lec-2 cells with membrane-inserted GM1, trigger significant inflammatory response in murine lung, and via an ATP-dependent signaling pathway stimulate mucin production in *MUC2*-transfected human cells [34,90]. After addition of *P. aeruginosa* flagellin onto polarized airway epithelial cells (16HBE), FliC was observed to co-localize with asialo-GM1 on the apical surface. TLR5 is predominantly expressed basolaterally, but after a prolonged incubation time, TLR5 was found mainly at the apical surface of 16HBE cells co-localized with flagellin [91]. Gangliosides were also reported to act together with TLR5 as co-receptors for *Salmonella* flagellin and trigger expression of the human antimicrobial peptide β-defensin-2 in Caco-2 cells [92]. An analysis of the role of asialo-GM1 and TLR5 in binding of flagellin and in flagellin-mediated signaling suggests that signaling down-stream of gangliosides is TLR-dependent and that the two flagellin receptors co-operate in activation of signaling pathways in epithelial cells [93]. Thus, the adhesive capacity of flagellin in combination with host ganglioside and TLR5 appears as an efficient mechanism in host defense in the intestine as well as lungs but may also enable bacterial migration.

Flagella have also been reported to function in bacterial symbiosis. Bacteria persist in multifaceted environments containing variable and dense bacterial populations, and they frequently cooperate in different aspects, like in the degradation of organic matter. Earlier reports have indicated that unidentified extracellular filaments are involved in the initiation of symbiosis based on nutritional cooperation, also called syntrophy, between the fermentative bacterium *Pelotomaculum thermopropionicum* and the methanogenic archaea *Methanothermobacter thermautotrophicus* [94]. Shimoyama and colleagues [95] observed putative flagella and fimbriae gene clusters in the genomes of *M. thermautotrophicus* and *P. thermopropionicum*. Extracellular filaments found in a monoculture of *P. thermopropionicum* were partially purified, and the 55 kDa major protein of the filaments was shown to be flagellin. Flagellum was shown to connect *M. thermautotrophicus* and *P. thermopropionicum* in cocultures. Purified recombinant FliC and FliD of *P. thermopropionicum* bound to cells of *M. thermautotrophicus* and to *Methanosaeta thermophile*, a syntrophic-association forming methanogen. In *M. thermautotrophicus*, methanogenesis was accelerated by FliD but not by FliC. Transcriptome analysis verified the results and revealed that FliD up-regulated more than 50 genes encoding e.g., methanogenesis-related enzymes, ATP synthase, and hydrogenases. The authors speculate that flagellum of *P. thermopropionicum* has a dual role in maintaining proximity between the two species and in synchronizing their metabolism.

## 5. Conclusions

**Figure 2:**
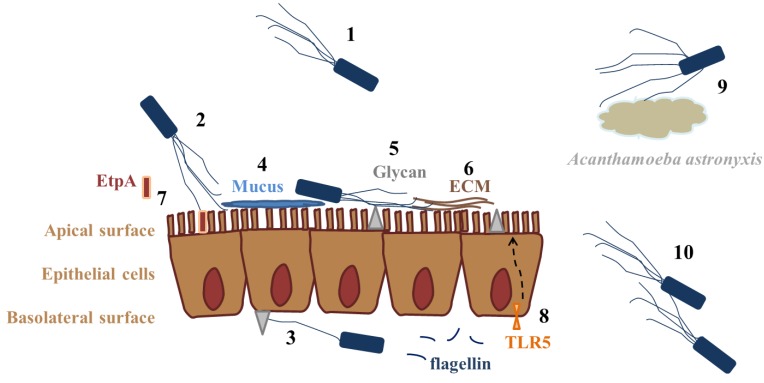
Summary of the bacterial flagellum as an adhesin. Flagellum can mediate bacterial adhesion to eukaryotic cells indirectly via motility (1), or by binding directly to epithelial cells either on apical (2) or basolateral surface (3). Flagellar target receptors include mucus and mucins (4), different glycans on cells or in mucus (gluconate, heparan sulfate proteoglycans, Lewis x glycotype, GM1, asialo-GM1, GD_1a_) (5), extracellular matrix (ECM) proteins (6), or bacterial-secreted EtpA, which in turn adheres to host cells (7). Toll-like receptor 5 (TLR5) is expressed mostly at the basolateral cell surface and binds FliC, inducing the host’s immune defense (8). FliC binding also induces TLR5 expression at the apical surface. In addition to various epithelia, flagella may also adhere to amoebae (9) or connect two bacterial species (10).

Flagella are involved in bacterial adhesion and invasion both indirectly, *i.e.*, by providing motility towards target cells and receptors, and directly by adhering to these targets. However, only a few receptors for flagellar adhesion have so far been convincingly revealed: gangliosides GM1 and GD_1a_, asialo-GM1, blood-group-antigen-related Lewis x glycotype, and heparan sulfate in the case of *P. aeruginosa*, and asialo-GM1, lipid rafts, gluconate, and EtpA in the case of *E. coli*. Flagellum-mediated adhesion and invasion have been mostly studied with whole flagella, or alternatively with purified flagellin (FliC) or flagellar cap (FliD) proteins. With the exception of the *N*- and *C*-terminal regions of FliC involved in innate immunity [12,13,14,15,16] and binding to the EtpA adhesin of ETEC [32], information on receptor-binding sites in flagella proteins is completely missing. Reports on other flagellar subunits, e.g., the hook or junction proteins, in adhesion, invasion, or innate immunity are totally lacking and would be important topics of further research. The existing knowledge on flagella adhesion is summarized in Figure 2.

Functions of flagella have mainly been studied in bacterial pathogens and from the viewpoint of bacterial virulence due to the various potential applications that exist for flagella and flagellin such as in vaccine development and diagnostics. For example, flagellin has been used for a generation of vaccines against *Salmonella enterica* serovar Paratyphi A, *P. aeruginosa*, and *E. coli* O157:H7 [96,97,98], and the colonization of EHEC in cattle was significantly decreased after vaccination with FliC [97]. Due to its high immunogenicity, flagellin has also been used as a vaccine adjuvant together with poorly immunogenic antigens, and FliC or anti-FliC antibodies have been used in diagnostics of e.g., melioidosis and inflammatory bowel disease [99]. Interestingly, flagellin has also been shown to suppress apoptosis and protect against radiation-related damage [100].

More recently, it has been noted that probiotic bacteria may also benefit from the flagellum, and that flagella can also be beneficial in symbiotic relationships between bacterial species. Thus many interesting aspects regarding the roles of flagella remain to be investigated in more detail.
